# Supplementary material for: Prebiotic Effect of Berberine and Curcumin Is Associated with the Improvement of Obesity in Mice
Source: Nutrients. 2021 Apr 24;13(5):1436. doi: 10.3390/nu13051436 (PMC8145536; doi:10.3390/nu13051436)
Supplement: Supplementary file 1 [file nutrients-13-01436-s001.zip › nutrients-1165974-supplementary.pdf]

Suppplementary Figure 1. Body weight evolution and cumulative food intake

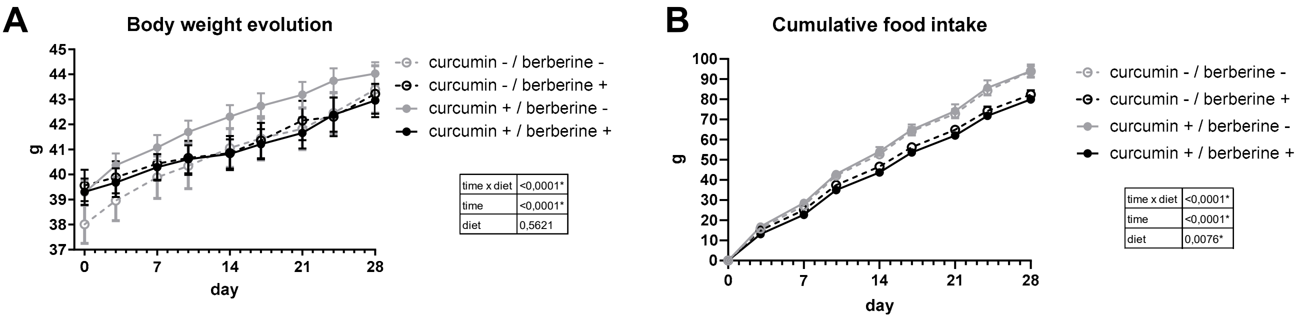

Ob/ob mice were fed a standard diet supplemented with or without berberine (bb) or curcumin (cc) for 4 weeks (A: n=9 for each group; B: n=3 for each group). *p<0.05 (two-way ANOVA).

Supplemental table S1. Primers used in RT-qPCR

Gene Symbol	Gene Name	Forward Primer	Reverse Primer
Ccl2	Chemokine (C-C motif) ligand 2	GCAGTTAACGCCCCACTCA	TCCAGCCTACTCATTGGGATCA
Ifng	Interferon gamma	AGCGGCTGACTGAACTCAGATTGTAG	GTCACAGTTTTCAGCTGTATAGGG
Il1β	Interleukin 1 beta	TCGCTCAGGGTCACAAGAAA	CATCAGAGGCAAGGAGGAAAAC
IL6	Interleukin 6	ACAAGTCGGAGGCTTAATTACACAT	TTGCCATTGCACAACTCTTTTC
Itgax (Cd11c)	Integrin alpha X	CAAAATCTCCAACCCATGCT	TGTGGTCAGCTCCACAGTTC
Lyz	Lysozyme	GCCAAGGTCTACAATCGTTGTGAGTTG	CAGTCAGCCAGCTTGACACCACG
Muc2	Mucin 2	ATGCCCACCTCCTCAAAGAC	GTAGTTTCCGTTGGAACAGTGAA
Ocln	Occludin	ATGTCCGGCCGATGCTCTC	TTTGGCTGCTCTTGGGTCTGTAT
Nox1	NADPH-oxidase	TTGGGTCAGCACTGGCTCTG	TGGCGGTGTGCAGTGCTATC
Pla2g2a	¨Phospholipase A2g2	AAGGATCCCCCAAGGATGCCAC	CAGCCGTTTCTGACAGGAGTTCTGG
Gcg	Preproglucagon	TGGCAGCACGCCCTTC	GCGCTTCTGTCTGGGA
Pyy	Peptide YY	GTTTGGACCAGTGGTGAAGA	TGCCCTCTTCTTAAACCAAACA
Reg3g	regenerating islet-derived 3-gamma	TTCCTGTCCTCCATGATCAAA	CATCCACCTCTGTTGGGTTC
Rpl19	Ribosomal protein L19	GAAGGTCAAAGGGAATGTGTTCA	CCTTGTCTGCCTTCAGCTTGT
Tjp1	Tight junction protein 1	TTTTTGACAGGGGGAGTGG	TGCTGCAGAGGTCAAAGTTCAAG
Tlr4	Toll-like receptor 4	CCCTCAGCACTCTTGATTGC	TGCTTCTGTTCCTTGACCCA
Tnf	Tumor Necrosis Factor	TCGAGTGACAAGCCTGTAGCC	TTGAGATCCATGCCGTTGG
Akkermansia spp.		CAGCACGTGAAGGTGGGGAC	CCTTGCGGTTGGCTTCAGAT
Bacteroides spp.		GGTGTCGGCTTAAGTGCCAT	CGGA(C/T)GTAAGGGCCGTGC
Bifidobacterium spp.		GATTCTGGCTCAGGATGAACGC	CTGATAGGACGCGACCCCAT
Lactobacillus spp.		AGCAGTAGGGAATCTTCCA	CACCGCTACACATGGAG
Total bacteria		ACTCCTACGGGAGGCAGCAG	ATTACCGCGGCTGCTGG

Supplementary Table S2. Colonic mRNA levels coding for proteins or peptides involved in the regulation of innate immunity, gut barrier and/or inflammation

Relative expression	**cc-/bb-**	**cc-/bb+**	**cc+/bb-**	**cc+/bb+**
Reg3g	1.00 ± 0.28[a]	0.11 ± 0.05[b]	0.45 ± 0.18[ab]	0.13 ± 0.04[b]
Lyz	1.00 ± 0.08	0.79 ± 0.08	1.65 ± 0.41	1.07 ± 0.15
Tjp1	1.00 ± 0.03	1.03 ± 0.04	1.07 ± 0.06	0.98 ± 0.03
Ocln #	1.00 ± 0.04[a]	0.90 ± 0.03[ab]	0.82 ± 0.03[b]	0.78 ± 0.04[b]
Tnf	1.00 ± 0.09	1.11 ± 0.08	2.08 ± 0.77	1.26 ± 0.16
Il1b §	1.00 ± 0.12	1.14 ± 0.10	1.55 ± 0.27	0.97 ± 0.89
Il6	1.00 ± 0.08	1.11 ± 0.08	1.18 ± 0.07	1.16 ± 0.09
Ifng	1.00 ± 0.12	2.12 ± 0.42	2.18 ± 0.42	2.14 ± 0.43

Ob/ob mice were fed a standard diet supplemented with or without berberine or curcumin for 4 weeks (n=9 for each group). #$p<0.05$ for curcumin effect, §$p<0.05$ for interaction effect (two-way ANOVA). Data with different superscript letters are significantly different at $p<0.05$ (Tukey post-hoc test).
